# Novel pharmacologic approach to enhance the epigenetic and immune priming effect of decitabine in patients with advanced non-small cell lung cancer

**DOI:** 10.1186/2051-1426-3-S2-P178

**Published:** 2015-11-04

**Authors:** Vamsidhar Velcheti, Nathan A Pennell, Sagar Rakshit, James Stevenson, Marc Shapiro, Francisco A Almeida, Ram Gurajala, Kurt Alex Schalper, Yogen Saunthararajah

**Affiliations:** 1 Cleveland Clinic, Cleveland, OH, USA; 2 Respiratory Institute, Cleveland Clinic, Cleveland, OH, USA; 3 Yale University, New Haven, CT, USA

## Background

DNA methyl transferase 1 (DNMT-1) is a key epigenetic enzyme in cancer cells which inactivates proliferation terminating genes and downregulates the expression of cancer related proteins and neoantigens by hypermethylation. Prolonged exposure of tumor cells to DAC is required for sufficient demethylation. Clinical use of decitabine to target DNMT-1 is restricted by its short in vivo half-life (about 10 minutes) due to rapid inactivation by the enzyme cytidine deaminase (CDA) present in tissues. Tetrahydrouridne(THU), an oral inhibitor of CDA can overcome the pharmacokinetic (PK) limitation.

## Hypothesis

Novel pharmacologic combination of THU with micro-doses of DAC can significantly increase half-life of DAC allowing sufficient interaction between DNMT-1 and decitabine to overcome epigenetic repression by DNMT-1. This could lead to enhanced expression of epigenetically silenced cancer-related proteins and antigens, attracting a robust immune response in the tumor milieu. Increased lymphocytic infiltrate in the tumor post the *immune priming* by decitabine can augment response to immune checkpoint inhibitor MPDL3280A, an anti PD-L1 antibody.

## Study design

**Figure 1:**
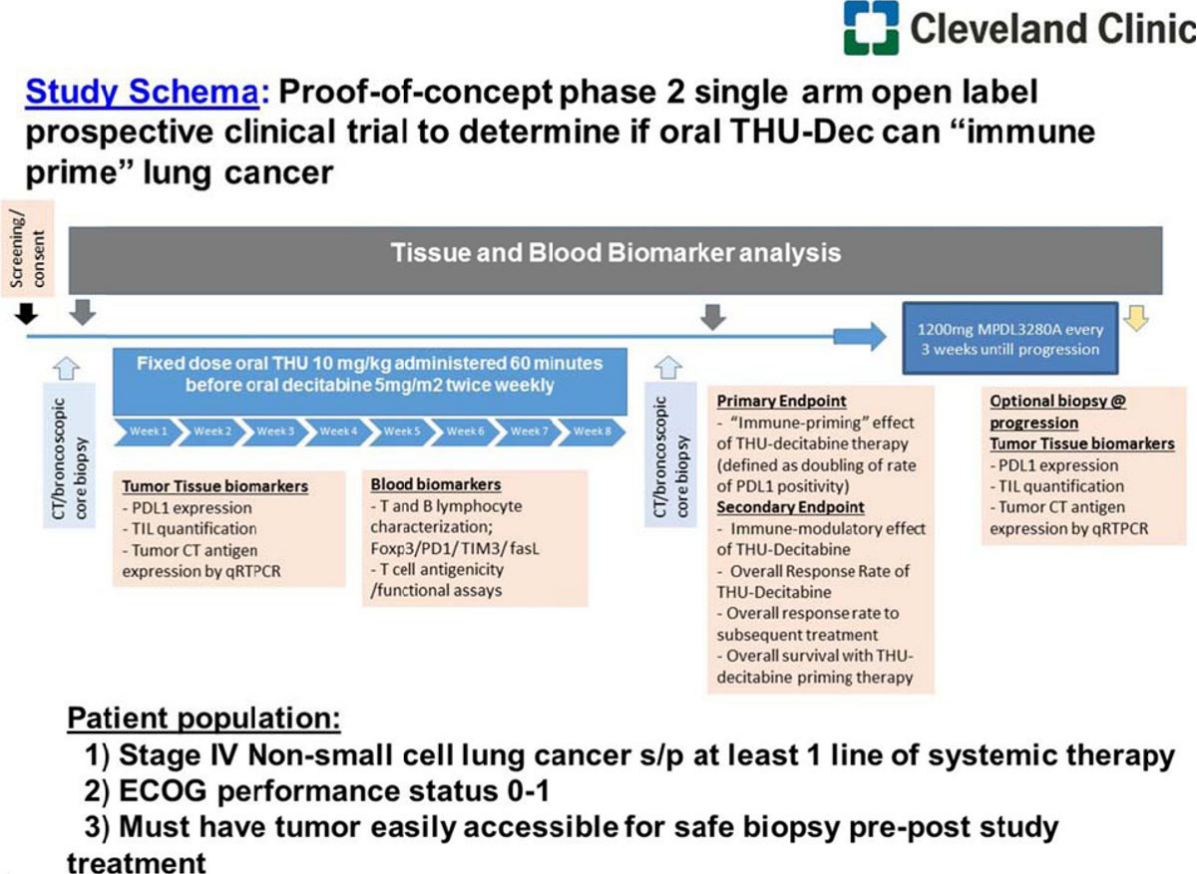


This is a proof-of-concept Phase II single arm open label prospective clinical trial in patients with advanced non-small cell lung cancer (NSCLC) with at least one prior chemotherapy (Figure 1).

## Methods

The study treatment would be DAC 0.002 mg/m^2^ and THU at a dose of 400 mg/m^2^ at a fixed dose administered orally 2X/week for 8 weeks followed by treatment with 1200mg MPDL3280A every 3 weeks until progression. The THU-DAC regimen for immune priming is selected based on ongoing Phase I clinical trial in nonmalignant disease, and IND enabling GLP toxicology studies. Doubling in the number of activated TILs in the tumor compared to pre-treatment biopsy will be considered evidence of immune induction by DAC. The trial will be conducted in two stages. The first stage will be an assessment of the immunologic effect of the combination. The second stage, which will occur only if THU-DAC is shown to have a significant priming effect, will assess the ability of the immunologic response to translate to clinical efficacy. Based on our previous studies we anticipate in advanced stage NSCLC approximately 25% patients will have high TILs and hypothesize that THU-DAC will increase that proportion to >50%. With 24 patients there will be at least 90% power to detect such a difference. Assuming the combination does indeed prime the immune system, stage 2 will be implemented and a two-stage accrual design will be used to assess clinical efficacy, with the 24 patients already accrued.

